# Kluyvera ascorbata: An Unusual Cause of Septic Shock in a Patient With Urothelial Cancer

**DOI:** 10.7759/cureus.51057

**Published:** 2023-12-25

**Authors:** Maria Akiki, Ruhma Ali, Asma Jamil, Jihad Slim, Richard Miller

**Affiliations:** 1 Internal Medicine, Saint Michael's Medical Center, Newark, USA; 2 Pulmonary and Critical Care Medicine, Saint Michael's Medical Center, Newark, USA; 3 Infectious Diseases, Saint Michael's Medical Center, Newark, USA

**Keywords:** multiorgan system failure, septic shock, urinary tract infection, urothelial cancer, kluyvera ascorbata

## Abstract

*Kluyvera ascorbata* is a gram-negative bacillus which is a rare cause of clinically significant infections in humans. We report a rare case of *K. ascorbata* infection causing septic shock in a patient with a history of urothelial cancer. After the antimicrobial susceptibility testing, the patient was successfully treated with ceftriaxone. Recognition of the disease-producing potential of this rare pathogen with prompt initiation of effective antimicrobial coverage is paramount for appropriate management in the adult immunocompromised population. To our knowledge, this is the first case report of septic shock secondary to *K. ascorbata* urinary tract infection.

## Introduction

*Kluyvera ascorbata* is a relatively new addition to the *Enterobacteriaceae* family and is rarely known to cause infections in adults [1]. This organism has been found in water, sewage, and hospital sinks and is recognized as part of the normal flora of the gastrointestinal, respiratory, and urinary tracts, but despite being part of the human flora, the organism's potential as a significant pathogen is rarely encountered in clinical practice [2]. A few case reports describe *K. ascorbata* causing appendicitis, cholangitis, and bacteremia; however, to the best of our knowledge, this is a unique case of a female with a history of urothelial carcinoma with a urostomy bag who had *K. ascorbata* urinary tract infection causing septic shock with multiorgan failure.

## Case presentation

A 66-year-old female with a past medical history of atrial fibrillation, congestive heart failure, and urothelial carcinoma diagnosed in December 2022 treated with neoadjuvant chemotherapy and later with radical cystectomy with ileal conduit urinary diversion with a urostomy bag placement in June 2023 presented to the emergency department (ED) with complaints of vomiting and decreased urine output from the urostomy bag for the past three days. The patient denied any fever, chills, or abdominal pain. On examination, her vitals showed temperature 98.7°F, pulse 30/min, respiratory rate (RR) of 22 breaths per minute (bpm), blood pressure 64/27 mm Hg, and saturating 94% on room air (RA). On physical examination, the patient looked fatigued and dehydrated. The abdomen was firm in the right lower quadrant, no tenderness was noted, and bowel sounds were present. Cardiac and respiratory examination was unremarkable. Electrocardiogram (ECG) showed sinus bradycardia. The patient was given intravenous fluids and pressor support for hypotension. She had acute kidney injury with elevated creatinine, high anion gap metabolic acidosis related to uremia, and acidosis. The initial laboratory results are shown in Table 1, Table 2, and Table 3.

**Table 1:** Complete metabolic panel, inflammatory markers, and lactic acid mmol/L: millimoles per liter; mg/dL: milligrams per deciliter; U/L: units per liter; g/dL: grams per deciliter; ng/mL: nanograms per milliliter

Complete metabolic panel, inflammatory markers, and lactic acid	Values	Reference range
Sodium	131	136-145 mmol/L
Potassium	5.3	3.5-5.3 mmol/L
Chloride	100	98-110 mmol/L
Carbon dioxide	16.8	20-31 mmol/L
Anion gap	14.2	6-19 mmol/L
Blood urea nitrogen	91	6-24 mmol/L
Creatinine	8.98	0.55-1.02 mg/dL
Glucose	104	70-140 mg/dL
Aspartate aminotransferase	9	10-36 U/L
Alanine transaminase	12	10-49 U/L
Alkaline phosphatase	157	46-116 U/L
Albumin/globulin ratio	1.2	0.8-2
Total protein	7.6	6.4-8.4 g/dL
Albumin	4.1	3.2-4.8 g/dL
Total bilirubin	0.5	02-1.2 mg/dL
Phosphorus	7.1	2.1-4.3 mg/dL
Magnesium	1.87	1.5-2.5 mg/dL
Calcium	9.5	8.6-19 mmol/L
C-reactive protein	7.6	0.0-0.8 mg/dL
Procalcitonin	2.11	0.0-0.5 ng/mL
Lactic acid	4.1	0-2 mmol/L

**Table 2:** Complete blood count uL: microliter; fL: femtoliter; g/dL: grams per deciliter; pg: picogram; %: percentage

Complete blood count	Values	Reference range
White blood cells	10.6	4.4-11 10^3^/uL
Red blood cells	4.15	3.9-5.03 10^6^/uL
Hemoglobin	11.7	12-15.5 g/dL
Mean corpuscular volume	36	81.6-98.3 fL
Mean corpuscular hemoglobin	86.6	27.5-33.2 pg
Mean corpuscular hemoglobin concentration	32.5	33.4-35.5 g/dL
Red cell distribution width	20.1	11.8-15.6%
Platelets	242	150-450 10^3^/uL
Mean platelet volume	8.3	7.4-11 fL

**Table 3:** Urinalysis pH: potential of hydrogen; HPF: high-power field; LPF: low-power field; EU/dL: equal to one milligram of urobilinogen per deciliter

Urinalysis	Values	Reference range
Bacteria	>30 many	None seen/HPF
Bilirubin	Negative	Negative, trace
Blood	2+	Negative
Casts	None seen	None seen/LPF
Clarity	Cloudy	Clear
Color	Yellow	Colorless, light yellow, yellow
Crystals	None seen	None seen/HPF
Glucose	Negative	Negative
Ketones	Trace	Negative
Leukocyte esterase	3+	Negative
Nitrite	Negative	Negative
pH	7.0	5-8
Proteins	3+	Negative
Red blood cells	3-10 few	0-5/HPF
Specific gravity	1.016	1.005-1.030
Squamous epithelial cells	None seen	None seen/LPF
Urobilinogen	1.0	0.2, <0.2 EU/dL
White blood cells	>25 many	0-2/HPF

Chest X-ray showed no focal consolidation, pneumothorax, or effusion. Computed tomography (CT) of the abdomen/pelvis without contrast showed no evidence of hydronephrosis. No renal or ureteral calculi were seen. Urinalysis showed many bacteria and white blood cells and leukocyte esterase +3. She was started on vancomycin and piperacillin-tazobactam. Over the next few hours, the patient was found to be more restless and confused. She was intubated for airway protection. Continuous venovenous hemofiltration (CVVH) was started due to refractory acidosis and oliguria. Urine culture was positive for *K. ascorbata* resistant to ampicillin. Antimicrobial susceptibility is shown in Table 4.

**Table 4:** Urine culture, Kluyvera ascorbata susceptibility

Urine culture, *Kluyvera ascorbata* susceptibility	
Ampicillin	Resistant
Ampicillin+sulbactam	Susceptible
Cefazolin	Susceptible
Cefepime	Susceptible
Ceftriaxone	Susceptible
Ertapenem	Susceptible
Gentamicin	Susceptible
Meropenem	Susceptible
Nitrofurantoin	Susceptible
Piperacillin+tazobactam	Susceptible
Tetracycline	Susceptible
Trimethoprim+sulfamethoxazole	Susceptible

Blood cultures were negative. Antibiotics were deescalated to ceftriaxone 1 gram daily for seven days. Over the course of the next few days, the patient was extubated and weaned off the dialysis. Her urine output improved, and her blood pressure improved without any further pressor requirement. The remainder of the hospital course was unremarkable. Inflammatory markers, white blood cell count, and lactic acid trended down as shown in Table 5.

**Table 5:** Inflammatory markers, white blood cell count, and lactic acid trend throughout the hospital course mg/dL: milligrams per deciliter; ng/mL: nanograms per milliliter; 10^3^/uL: 10^3^ per microliter; mmol/L: millimoles per liter

	On admission (10/17/2023)	10/18/2023	10/20/2023	10/29/2023
C-reactive protein (mg/dL)	7.6	10.1	4.9	<0.4
Procalcitonin (ng/mL)	2.11	2.11	0.38	0.07
White blood cell count (10^3^/uL)	10.6	11	6.8	6.5
Lactic acid (mmol/L)	4.1	3.7	1.6	0.9

The patient was discharged with outpatient follow-up with urology and nephrology.

## Discussion

In 1936, Kluyver et al. proposed the existence of a distinct group of fermentative bacteria within the *Enterobacteriaceae* family [2]. After that, Asai et al. identified a group of organisms that they matched Kluyver's proposition and suggested the genus name *Kluyvera* [2]. However, few years later, the same researchers recommended eliminating the genus identification, believing that the organism was phenotypically similar to *Escherichia* [2]. *Kluyvera* was only recognized as a distinct genus in 1981 when Farmer and colleagues redefined it through the use of biochemical and DNA-DNA hybridization methods. This redefinition led to the acknowledgment of more *Kluyvera* strains and the emergence of additional clinical insights from infection reports associated with this organism [3]. *Kluyvera* is a flagellated and motile gram-negative bacillus that falls within the family *Enterobacteriaceae *[3]. Thriving in standard culture media, *Kluyvera* colonies closely resemble those of *Escherichia* but are citrate positive [3]. While no specific virulence factor has been pinpointed, the organism shares similarities with other *Enterobacteriaceae*, having a lipopolysaccharide complex and surface antigens that could potentially grant it virulence [3]. The genus comprises four species: *K. ascorbata*, *K. cochleae*, *K. georgiana*, and *K. cryocrescens* [3]. Among these, *K. ascorbata* is the most frequently isolated in clinical specimens [3]. *Kluyvera* is part of the normal flora of the human gastrointestinal tract, typically associated with low bacterial counts, potentially explaining its rarity in clinical infections [2]. *Kluyvera* is recognized as a colonizer of the respiratory tract [3]. *Kluyvera* is also found in water, soil, sewage, and healthcare environments [2].

Since 1980, numerous clinically significant infections attributable to *Kluyvera* have been documented [2]. In 2001, Sarria et al. conducted the most extensive retrospective study to date on the clinical manifestations of *Kluyvera*-related infections [2]. The spectrum encompassed gastroenteritis, acute pancreatitis, bacteremia, wound infection, urinary tract infection (UTI), pyelonephritis, acute cholecystitis, peritonitis, mediastinitis, infected urethrorectal fistula, and soft tissue infection [2]. UTI caused by *Kluyvera* can vary from uncomplicated cystitis to pyelonephritis accompanied by septic shock. Sarria et al. documented a case of non-complicated lower UTI in a previously healthy 36-year-old patient [2]. Alfreijat described a case of UTI and severe sepsis secondary to *K. ascorbata* that was complicated by cardiac arrhythmia pretty similar to our case; however, the patient did not require vasopressors [4]. Inoue et al. described a case of UTI secondary to *K. intermedia* with bacteremia and septic shock [5]. There have been no reported cases resembling our case, of complicated UTI with septic shock attributed to *K. ascorbata*. Sarria et al. study revealed that effective antimicrobial agents against the majority of *Kluyvera* strains included third-generation cephalosporins, aminoglycosides, and fluoroquinolones [2]. Moreover, resistance was observed against extended-spectrum penicillins, ampicillin, and first- and second-generation cephalosporins [2]. In our case, *K. ascorbata* was susceptible to third-generation cephalosporins, and the patient was effectively treated with ceftriaxone.

## Conclusions

*K. ascorbata* is gaining recognition as an unusual yet clinically significant pathogen, manifesting in diverse disease forms. Its presence in the clinical setting should not be neglected. *K. ascorbata* has the potential to be life-threatening especially in immunocompromised patients. Clinicians must remain vigilant to its possible pathogenic role and administer adequate antimicrobial therapy to prevent serious complications.
